# New tools for experimental diabetes research: Cellular reprogramming and genome editing

**DOI:** 10.3109/03009734.2016.1149529

**Published:** 2016-03-16

**Authors:** Timo Otonkoski

**Affiliations:** 1 Research Programs Unit, Molecular Neurology and Biomedicum Stem Cell Center, University of Helsinki, Children’s Hospital,; 2 Children’s Hospital, University of Helsinki and Helsinki University Hospital, Helsinki, Finland

**Keywords:** Diabetes, islet transplantation, molecular biology

## Abstract

Isolated human islets are a rare and precious material for diabetes research. However, their availability is limited, and it is impossible to obtain them from patients with specific genotypes. Human pluripotent stem cells provide an alternative. Induced pluripotent stem cells can be generated from any individual’s somatic cells and differentiated into pancreatic cells. Currently, this approach is limited by the immaturity of the islet-like cells derived from stem cells. However, this approach can already be used to model developmental defects, and the possibilities for studying insulin secretion are continuously improving. In addition, genome editing using the CRISPR/Cas9 technology provides powerful possibilities to study the impact of specific genotypes. The same technology can also be used for transcriptional regulation in order to improve the functional maturation of stem cell-derived islets. These tools are today becoming available for tomorrow’s translational diabetes research.

In order to understand diabetes, you need to know how the insulin factory operates. This fact has gradually become generally accepted among diabetes researchers. Claes Hellerström understood it from the beginning of his research career. It is intriguing that his first article that can be found in PubMed, written together with Bo Hellman in 1959, deals with diurnal changes in the functional activity of pancreatic islets as reflected by changes in nuclear size (1). This same topic is currently under intensive investigation, as evidenced for example by the annual IGIS Symposium theme in 2015, ‘The islet and metabolism keep time’ (2). This example demonstrates how central research questions have been in the minds of far-sighted investigators for a long time, even if the means to address them are continuously evolving.

One of the most important research themes of Claes Hellerström was pancreatic islet growth and mass regulation. These studies were greatly enhanced when Claes, together with Norbert Freinkel, developed a method for the efficient generation of rat islets from late fetal pancreatic tissue, based on the spontaneous budding of islets from epithelial cells (3). The same approach was later applied to human fetal pancreatic tissue, enabling for the first time controlled experimental studies of human islet development and growth (4). At this time, in approximately 1985, I had the chance to visit the BMC in Uppsala for the first time and start learning from Claes and his team in a most hospitable and generous atmosphere. It was evident that understanding of the molecular mechanisms controlling beta cell expansion and maturation could provide crucial information for the development of new therapeutic strategies for diabetes. Twenty years later we have learned a lot, but we are still on the same learning road, addressing the same basic questions with new approaches.

Although rodent animal models are invaluable tools also for beta cell biology, it is obvious that there are important differences between mouse and human physiology, highlighting the need for human islet cell models. Moreover, rodent islet cells cannot be used for therapeutic purposes in humans. This is why a lot of effort has been put into the development of human islet isolation from cadaveric donors for clinical islet transplantation. As a result, isolated human islets have become an important resource also for research. This material is problematic, however, because of its limited availability and high variability due to often poor viability of the cells. Human fetal pancreatic tissue is a very valuable resource, but ethical issues and practical reasons severely limit its availability. Alternative human islet and beta cell models are thus needed. Pluripotent human stem cells provide an attractive possibility (5). In this review, I briefly outline the progress and prospects of using pluripotent stem cells, nuclear reprogramming, and genome editing for studying human beta cell biology and disease.

## Human pluripotent stem cells as biomedical research tools

A lot of excitement, and also excessive ‘hype’, was created by the seemingly endless regenerative medical possibilities of human embryonic stem cells (hESC) when they were first described in 1998 by Thomson and colleagues (6). Type 1 diabetes has been at the top of the list of diseases that would be soon cured with these ‘magical’ cells. For anyone who understands fundamental biological principles it was clear that this would not be an easy goal. Human embryonic stem cells are wonderful research tools, representing pre-gastrulation stage primitive embryonic cells. When allowed to differentiate spontaneously, they do develop into all lineages derived from the three embryonic germ layers. However, they do this in an uncoordinated and stochastic manner, resulting in the formation of continuously growing teratomas when transplanted into rodents. The hESC-derived teratomas contain highly differentiated organ structures, demonstrating that embryonic inductive events must have occurred in a physiological manner. Human embryonic stem cells thus provide a unique model to study human development and to generate desired cell and tissue types.

One of the obvious main problems of hESC is the fact that their derivation is dependent on the loss of a viable human embryo. Although these are surplus embryos from infertility treatments, this has been a major ethical limiting factor against the use of hESC in many countries. The situation changed dramatically with the introduction of pluripotent reprogramming, allowing the derivation of hESC-like induced pluripotent stem cells (iPSC) from easily accessible somatic cells, such as skin fibroblasts and blood cells (7,8). It is now clear that completely reprogrammed iPSC are as pluripotent as hESC, irrespective of the cell type of origin (9). Nuclear reprogramming may allow the derivation of even more primitive stem cell stages, which may potentially be beneficial for the derivation of some cell lineages that are otherwise difficult to obtain (10).

## The long and winding road of pancreatic islet cell differentiation from the pluripotent stem cell

Once the essential principles of primary lineage commitment had been worked out, robust and reproducible methods could be developed for the derivation of specific cell lineages from the pluripotent cells, following principles learned mainly from mouse developmental biology. However, controlled differentiation of pluripotent stem cells into fully functional mature cell types remains a major challenge. This is true also for the pancreatic endocrine cells, despite the recent remarkable progress.

The first report of spontaneously differentiated insulin-expressing mouse embryonic stem cells as therapeutic agents for diabetes, by Soria et al., appeared in 2000 (11). At this time, researchers did not fully appreciate the complexities of recapitulating organ development in a dish. Later, the sequential developmental stages observed *in vivo* were mimicked *in vitro* by providing the necessary key growth factors and signals. A crucial step was the establishment of a method for the derivation of definitive endoderm using a high concentration of activin A together with Wnt3a (12). After definitive endoderm specification, retinoic acid signaling was found to increase PDX1 levels, achieving the posterior foregut stage, which was further improved by activating FGF signaling and inhibiting sonic hedgehog signaling pathway (13–16). Induction of the pro-endocrine program through activation of NEUROG3 was achieved by downregulation of Notch signaling with gamma-secretase inhibitors. Differentiation towards pancreatic lineage and blockade of hepatic specification was improved by inhibiting BMP signaling (16,17). As a result of this development, up to 25% of C-peptide-positive cells were yielded at the final stage. However, most of these cells were polyhormonal and did not show robust insulin secretion in response to a glucose challenge. Important next steps were needed to generate proper beta cell progenitors that were able to differentiate into monohormonal cells. Rezania and colleagues showed that NKX6.1 induction by activation of the PKC pathway before NEUROG3 expression resulted in monohormonal insulin-expressing cells, while the NKX6.1-negative cells yielded polyhormonal and glucagon-positive cells (18). Similarly, BMP inhibition with Noggin combined with EGF and nicotinamide was reported to induce NKX6.1 robustly in different human pluripotent stem cell (hPSC) lines, resulting in pancreatic progenitors that gave rise to monohormonal endocrine cells after maturation *in vivo* (19).

Currently, robust protocols are available that can generate large numbers of islet-like cellular aggregates from human pluripotent stem cells, containing monohormonal insulin- and glucagon-expressing cells. The insulin-expressing cells express beta cell markers, have gene expression profiles close to adult beta cells, show functionality by secreting insulin in response to glucose in static conditions, and restore normoglycemia after transplantation into diabetic mice (20,21). However, further characterization of these cells shows that they are not fully mature in terms of dynamic responses of insulin secretion or intracellular calcium fluxes in response to glucose.

The challenge of pluripotent stem cells is that they are truly primitive stem cells and it is difficult to guarantee that they completely lose their pluripotent capacity and become exclusively committed to the desired lineage. A theoretically attractive alternative is based on the idea of direct conversion of somatic cells into lineage-specific expandable progenitors that can then be further differentiated into mature cells. There are some examples suggesting that this strategy may result in more functional cell types, for example hepatocytes, as compared with differentiation starting from the pluripotent stage (22). It is, however, likely that the conversion of cell fate involves a brief stage of pluripotency (23). Recently, Hebrok, Ding and colleagues showed that the direct conversion strategy can be successfully applied to generate robustly expandable pancreatic progenitors from human fibroblasts. These cells maintained their capacity to differentiate into functional insulin-producing cells capable of maintaining normoglycemia in diabetic mice (24). This is a highly promising example of the possibilities of cellular reprogramming in the development of therapeutic cells.

## Genome editing changes the scene

Development of custom-engineered nucleases for the introduction of site-specific DNA double strand breaks (DSBs) has greatly increased the feasibility of genome editing. Particularly, the CRISPR/Cas9 technology has rapidly gained popularity due to its high efficiency and versatility. Human pluripotent stem cells are ideal for genome editing, thanks to their unlimited life-span. Using this method, it is possible to generate DSBs at precisely defined target sites. The DSBs become rapidly corrected through the error-prone non-homologous end joining (NHEJ) mechanism which leads to frequent small insertions or deletions, allowing rapid generation of genetic knock-outs. Alternatively, if a donor template is simultaneously introduced in the cell, precise homology-directed repair may occur, resulting in the insertion of the desired sequence. This allows the generation or correction of specific mutations (25).

Using genome editing through CRISPR/Cas9, it is now possible to study the functional impacts of specific disease-associated mutations in an isogenic context, comparing cells with the same genetic background, discordant only for the specific mutation of interest. This greatly enhances the possibilities for reliable experimental settings. We and others have shown that the genetic background of individual donors of iPSC has a major impact on the differentiation behavior of the stem cells (9,26). As a result, it is often difficult to tell whether observed differences are due to interindividual variability or to the specific genetic variant of interest. This may be addressed by studying large enough collections of healthy control versus patient cells, but the possibility to confirm the results with engineered isogenic cell lines should markedly increase the power of the approach.

The CRISPR/Cas9 technology can be used also for other purposes than genome editing. The nuclease Cas9 can be mutated to become catalytically inactive (dead Cas9, dCas9) and fused with factors that mediate transcriptional activation or repression (27,28). These artificial transcription factors can be guided by specific guide RNAs into transcription regulatory areas (e.g. promoters) to control the transcriptional activity of desired genes. The system can also be made conditionally activatable by drugs, through the use of dihydrofolate reductase destabilization domain (stabilized by trimethoprim, TMP) or the doxycycline-inducible tet-on promoter. Using the combination of these two chemically regulatable transcriptional activator systems, we showed that it is possible sequentially to activate master genes controlling the differentiation of definitive endoderm and pancreatic progenitors (29). This strategy is likely to be a powerful tool for many purposes. The challenge of *in vitro* functional maturation of stem cell-derived beta cells can now be addressed by specifically activating (or repressing) specific genes known or expected to be crucial for the maturation process.

## Tomorrow’s translational diabetes research

**Figure 1.**
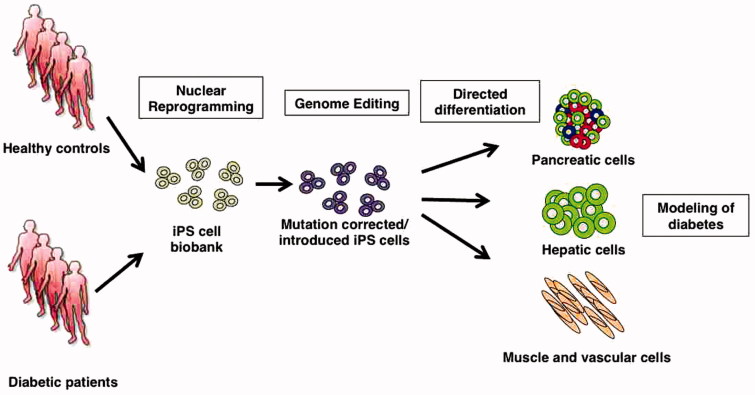
The use of cellular reprogramming and genome editing for experimental diabetes research. Induced pluripotent stem cell (iPSC) collections are made from cohorts of patients with genotypes of interest. The iPSC are then differentiated into relevant cell types, such as pancreatic islet cells, hepatocytes, or any other cell of interest. Functional comparisons are made between patient and control cells, and the findings can be confirmed by specifically editing the mutations of interest.

The approaches described above may revolutionize the possibilities for functional validation of specific genetic variants in cell types that are particularly relevant for the disease of interest. In the case of diabetes, it is now possible to create a substitute for the elusive endogenous patient beta cells. Collections of iPSC lines can be created from groups of individuals carrying a particular genotype and differentiated into pancreatic islet-like cells that can be compared with similar cells derived from healthy controls. The results can be further validated by engineering the specific mutation in healthy control cells or correcting it in the patient cells (Figure 1). Notably, the mutations can equally well be in protein coding or non-coding regions, or even in distal regulatory elements (30). The possibilities seem endless. However, one should remember that currently it is still not possible to create a complete ‘replica’ of the mature functional beta cell that would recapitulate all the complex signaling responsible for physiologically regulated insulin secretion. Nevertheless, the progress in this field has been extraordinarily rapid, making it realistic to estimate that this goal keeps getting closer and closer.

The translational researcher of tomorrow will have a completely new toolbox at hand to gain understanding in the pathophysiological mechanisms of complex diseases, such as diabetes mellitus. Cellular reprogramming makes it possible to generate *in vitro* models of the tissues of interest, based on the exact genotypes linked with the disease. In addition to the pancreatic endocrine cells, also important insulin target tissues can be generated, such as liver, muscle, or cardiovascular cells. Experiments can then be planned to pinpoint the molecular pathways and their functional outcomes that are affected by the specific genotypes (Figure 1). In addition to obvious functions, such as insulin secretion and the target cell responses, a wide range of additional parameters can equally well be studied, such as development and growth, intercellular communication, and cell death. Naturally all of this can be studied, both at detailed pathway-specific molecular level and at the global transcriptomic, epigenetic, or proteomic level.

Claes Hellerström did not live to see the evolution of cellular reprogramming and genome editing for translational medical research. Without doubt, he would have actively welcomed and promoted these possibilities which are taking us step by step closer to the goals that were most important to him: detailed understanding of pancreatic beta cell growth and function, and application of this knowledge towards therapy.
